# Rivaroxaban does not influence hemorrhagic transformation in a diabetes ischemic stroke and endovascular thrombectomy model

**DOI:** 10.1038/s41598-018-25820-y

**Published:** 2018-05-09

**Authors:** Feng-Di Liu, Rong Zhao, Xiao-Yan Feng, Yan-Hui Shi, Yi-Lan Wu, Xiao-Lei Shen, Ge-Fei Li, Yi-Sheng Liu, Ying Zhao, Xin-Wei He, Jia-Wen Yin, Mei-Ting Zhuang, Bing-Qiao Zhao, Jian-Ren Liu

**Affiliations:** 1 Department of Neurology, Shanghai Ninth People’s Hospital, Shanghai Jiao Tong University School of Medicine, Shanghai, China; 2 Department of Neurology, Wuxi No. 2 People’s Hospital, Wuxi, Jiangsu China; 3 State Key Laboratory of Medical Neurobiology, Institutes of Brain Science, Collaborative Innovation Center for Brain Science and School of Basic Medical Sciences, Fudan University, Shanghai, China

## Abstract

Managing endovascular thrombectomy (ET) in diabetic ischemic stroke (IS) with novel anticoagulants is challenging due to putative risk of intracerebral hemorrhage. The study evaluates increased hemorrhagic transformation (HT) risk in Rivaroxaban-treated diabetic rats post ET. Diabetes was induced in male Sprague-Dawley rats by intraperitoneal injection of 60 mg/kg streptozotocin. After 4-weeks, rats were pretreated orally with 30 mg/kg Rivaroxaban/saline; prothrombin time was monitored. IS and ET was induced after 1 h, by thread-induced transient middle cerebral artery occlusion (tMCAO) that mimicked mechanical ET for proximal MCA occlusion at 60 min. After 24 h reperfusion, infarct volumes, HT, blood-brain barrier (BBB) permeability, tight junction at peri-ischemic lesion and matrix metalloproteinase-9 (MMP-9) activity was measured. Diabetic rats seemed to exhibit increased infarct volume and HT at 24 h after ET than normal rats. Infarct volumes and functional outcomes did not differ between Rivaroxaban and diabetic control groups. A significant increase in HT volumes and BBB permeability under Rivaroxaban treatment was not detected. Compared to diabetic control group, neither the occludin expression was remarkably lower in the Rivaroxaban group nor the MMP-9 activity was higher. Together, Rivaroxaban does not increase HT after ET in diabetic rats with proximal MCA occlusion, since Rivaroxaban has fewer effects on post-ischemic BBB permeability.

## Introduction

Atrial fibrillation (AF) is the most common sustained cardiac arrhythmia encountered clinically^1,2^. In 2050 there will be an estimated 72 million AF patients in Asia^3^, with a 5-fold higher risk of ischemic stroke (IS). Embolic cerebral infarction associated with AF can be prevented by oral anticoagulants^4^. But some anticoagulants such as warfarin increase the risk of intracranial hemorrhage^5^. The introduction of direct thrombin (Dabigatran)^6^ and factor Xa inhibitors (Rivaroxaban and Apixaban), known as non-vitamin K antagonist oral anticoagulants (NOACs), are a major breakthrough because they are non-inferior or even superior to warfarin for prevention of stroke and bleeding side effects without monitoring^7,8^.

NOACs carry a substantially lower risk of intracranial hemorrhage (ICH) than warfarin. A mouse model suggests that doses of Rivaroxaban 30-fold higher than those administered clinically are required to induce intracranial hemorrhage^9^. Patients with NOAC-associated ICH had smaller ICH volumes and better clinical outcomes compared with warfarin-associated ICH in a small multicenter study^10^. Recent studies also show no increased risk of hemorrhagic transformation (HT) after IS in normal rats or mice under NOAC treatment, regardless of whether they received tissue-type plasminogen activator treatment or not^11,12^. But, the most recent clinical studies suggest there are some bleeding risks with Rivaroxaban. Compared to other NOACs and vitamin K antagonists, a retrospective cohort study showed Rivaroxaban was associated with increased gastrointestinal bleeding^13^. Among NOACs, Rivaroxaban patients have a significantly higher risk of major bleeding than Apixaban^14^ and Dabigatran patients^15,16^. So, the risk of HT with Rivaroxaban may be a concern.

Diabetes is an increasingly growing epidemic; patients with diabetes are at a 2–6-fold increased risk of stroke^17^. 70% patients with recent stroke have overt diabetes or pre-diabetes distinguished by impaired fasting glucose or impaired glucose tolerance^18^. In addition, diabetes and hyperglycemia predict early neurological deterioration following IS and patients have poorer prognosis than the non-diabetic population^19^. Pre-stroke hyperglycemia causes infarct expansion, develops greater HT and results in worse clinical outcome due to damage of blood-brain barrier (BBB)^17,20,21^.

Although intravenous recombinant tissue plasminogen activator (rt-PA) is the standard treatment for acute IS, the narrow time window and strict exclusion criteria limit its applications^22^. In 2015, a series of clinical trials showed that endovascular thrombectomy (ET) had higher rate of recanalization and good clinical outcome^23–25^. However, diabetes and anticoagulants are also independent predictors for HT after ET, which affect patients’ outcomes^26,27^. Hitherto, despite the increased risk of HT after ET in patients with diabetes, no data concerning the effects of Rivaroxaban and diabetes on ET in ischemic stroke have been published. Clinical study of NOACs in this subgroup of patients is challenging owing to high technique requirements and perioperative complication risk. The present study used an experimental diabetes and ET model of IS in rats to assess the effect of anticoagulation with Rivaroxaban on the risk of HT and its neurological outcome.

## Materials and Methods

### Animals

The study was approved by the Animal Care and Experiment Committee of the Ninth People’s Hospital affiliated to the School of Medicine, Shanghai Jiao Tong University [approval No. HKDL(2016)261] and all methods were performed in accordance with the relevant guidelines and regulations. Ninety-six male Sprague-Dawley rats (6–8 weeks, 200–250 g, Slac laboratory animal, Shanghai, China) received water and food without restrictions and were housed in a 12:12 h light/dark cycle at 24 °C with appropriate humidity. Surgeries were performed under isoflurane anesthesia; the suffering was minimized. The ARRIVE guidelines increase the reproducibility and quality of the data^28^.

Ninety-six rats were divided into 4 groups: Normal group, 18 normal rats (non-diabetic) underwent 1 h transient middle cerebral artery occlusion (tMCAO); Control group, 36 diabetic rats underwent 1 h tMCAO; Rivaroxaban group, 36 diabetic rats received Rivaroxaban treatment 1 h before tMCAO; Sham group, 6 diabetic rats underwent similar operation although with only superficial filament insertion into proximal internal carotid artery.

### Type 1 diabetes models

Seventy-eight rats fasted overnight. Diabetes was induced by a single intraperitoneal injection of 60 mg/kg streptozotocin (STZ, Sigma, St Louis MO, USA) dissolved in pH 4.5 citrate buffer, to a final concentration of 10 mg/ml. Diabetes was confirmed 3 days after STZ administration by tail pricking to analyze the blood glucose levels. Animals with random blood glucose concentrations >11.0 mmol/L were considered diabetic and included in the study^29^.

### Drug administration

Four weeks post-establishment of diabetic models, 36 rats received 30 mg/kg Rivaroxaban (Bayer HealthCare AG, Wuppertal, Germany) via gastric gavage 1 h before tMCAO. This dose was determined according to previous studies and a preliminary experiment that tested 3, 10, and 30 mg/kg doses of Rivaroxaban. 30 mg/kg dosage increased the prothrombin time^27^ by 2- to 3-fold, and this effect was sustained for >4 h^9,12^. Micronized Rivaroxaban was dissolved in saline at 3 mg/mL. To eliminate effects of trauma caused by the gastric gavage, remaining animals received 10 mL/kg saline via gastric gavage 1 h before operation. The gastric gavage was introduced under brief 3% isoflurane anesthesia^30^.

### Endovascular thrombectomy (ET) models

Because the tMCAO model showed abrupt cerebral blood reperfusion after the thread was mechanically pulled out, it could be used as an ET model^31^.

All rats were anesthetized with a mixture of nitrous oxide/oxygen/isoflurane (69%:30%:1%) during surgical preparation with an inhalation mask. Focal cerebral ischemia and ET was induced using the tMCAO model as described previously^11,32,33^. The rats’ body temperature was monitored and maintained at 37.0 ± 0.5 °C by a heating pad during the surgical procedure. The right middle cerebral artery (MCA) was occluded by inserting a 4–0 surgical nylon filament through the common carotid artery. Regional cerebral blood flow (CBF) was continuously monitored under general anesthesia with the probe located 1 mm posterior to the bregma and 5 mm from the midline of a hemisphere, an area comprising the territory of MCA, by laser-Doppler-flowmetry (Periflux system 5000, Perimed, Järfälla, Sweden). A significant drop in CBF indicated successful proximal occlusion of the MCA by the thread head. A similar degree of reduction in CBF was achieved in both Control and Rivaroxaban-treated rats. After 60 min of tMCAO, the nylon thread was gently removed (“endovascular mechanical thrombectomy”). CBF was restored after reperfusion and appeared to be similar in both groups. The anesthesia of all groups lasted until closure of the skin wound on the neck. Buprenorphine Hydrochloride (TIPR Pharmaceutical Responsible Co., Ltd, Tianjin, China) 0.05 mg/kg was injected subcutaneously to relieve post-surgical pain^34^. The animals were returned to their cages immediately after they had regained consciousness. Rats that died owing to subarachnoid hemorrhage, anesthetic accident or difficulty in inserting the thread were excluded.

### Coagulation status

The rats’ coagulation status was determined by measuring Prothrombin time^27^ with CoaguChekXS (Roche Diagnostics GmbH, Mannheim, Germany). PT was monitored before inserting and after removing the nylon filament.

### Neurological deficit

Neurological deficit was assessed using the Bederson and Garcia scales scores^35^ 24 h post-reperfusion and 12 rats per group from the Control and Rivaroxaban group were included. Bederson scale: 0 = no deficit; 1 = flexion of forelimb; 2 = consistent reduction in resistance to lateral push toward the paretic side; 3 = circling toward the paretic side; 4 = fall down to the paretic side; 5 = unable to move spontaneously; 6 = dead. The 18-point Garcia scale includes testing spontaneous activity, symmetry in the movement of four limbs, forepaw outstretching, climbing, body proprioception and vibrissae touch. The observation was performed by a person blinded to the experimental groupings. Rats that died within the observation period were given 0 points in the Garcia scale score.

### Preparation of sections and tissue samples

24 h post-ET surgery, animals were anesthetized and transcardially perfused with phosphate-buffered saline (PBS; pH 7.2). The whole brain was removed, 3-mm-thick coronal sections were cut, and stained with 2% 2,3,5-triphenyltetrazoliumchloride (TTC, Sigma, St Louis, MO, USA). Six rats in the Normal, Control, Rivaroxaban group determined the infarct volume that was calculated by the standard method^36,37^ with an image analysis program (ImageJ, version 1.37 V, NIH, Bethesda, MD, USA), thus correcting edema. The infract volume was expressed as a percentage of the whole contralateral hemisphere.

Infarct volume (%) = [(total cortex volume in the contralateral cerebral hemisphere − noninfarcted cortex volume in the ipsilateral cerebral hemisphere)/total cortex volume in the contralateral cerebral hemisphere] × 100%^37^.

### Quantification of HT

HT volume was measured as follows: a previously published spectrophotometric method measuring hemoglobin concentration and photograph scoring^12^. However, the hemoglobin concentration results were below the sensitivity of the method, and hence, were not presented. In order to evaluate the HT volume quantitatively, we used image analysis as an alternative^38^. In brief, regions of hemorrhage were visually identified and outlined manually, and areas were then integrated to yield total volumes. The unstained coronal sections were imaged every 3 mm and evaluated by two independent raters that were masked to group assignment. ImageJ (version 1.37 V, NIH, Bethesda, MD, USA) was used to analyze HT volumes.

Hemorrhage score ranged from 0–to 4: 0 = no hemorrhage; 1 = dispersed individual petechiae; 2 = confluent petechial; 3 = small diffuse hemorrhage or hematoma; 4 = large diffuse hemorrhage or hematoma^39^. Each of the Normal, Control, and Rivaroxaban group used 12 rats to evaluate HT score.

### BBB permeability for Evans Blue (EB)

To quantify post-ischemic BBB permeability, 6 animals each from the Control and Rivaroxaban groups were operated as described above with ET surgery. Animals were briefly reanesthetized 24 h after reperfusion and 2% EB (Sigma, St Louis MO, USA) in saline (4 mL/kg) was injected into the femoral vein. After 2 h, rats were transcardially perfused with PBS and the ipsilateral hemisphere was removed and homogenized in 3 mL formamide. After extraction in 60 °C water for 24 h, the samples were centrifuged at 2000 × g for 20 min. EB concentration of the supernatant was determined at 620 nm (Thermo Scientific BioMate 3S) and quantified as µg of EB/mg tissue using a standard curve^40^.

### Gelatin zymography and western blot

For western blot and gelatin zymography, 6 rats each from the Control, Rivaroxaban, and Sham groups were treated as above. 24 h post-reperfusion, the rats were anesthetized by intraperitoneal injection of chloral hydrate (400 mg/kg) and transcardially perfused with PBS. Brains were removed quickly and divided into ipsilateral–peri-ischemic and contralateral–nonischemic hemispheres. Each hemispheric brain was frozen immediately in dry ice and stored at −80 °C until use.

Gelatin zymography was performed using the frozen brain tissue. Frozen brain samples were homogenized in 10× volume lysis buffer (150 mmol/L NaCl, 1% SDS, 0.1% deoxycholic acid, and 50 mmol/L Tris-HCl; pH 7.4) containing protease inhibitors. The supernatant was collected at 9000 × g for 15 min at 4 °C. The total protein concentration was determined by Bicinchoninic acid kit (Sigma, St Louis MO, USA). The activity of matrix metalloproteinase-9 (MMP-9) in each sample was measured using a gelatin-zymography kit (Xinfan Biotechnology, Shanghai, China) according to the manufacturer’s instructions. Brief, each sample containing 20 μg protein was diluted with the homogenizing buffer in the kit, mixed with an equal volume of sample buffer, and electrophoretically resolved for 2 h. The gels were washed and incubated for 24 h in incubation buffer at 37 °C and stained with Coomassie blue. Quantitative densitometric analysis was performed with ImageJ software.

For occludin levels, 7 μg of total protein extract was electrophoresed on 12% polyacrylamide gels and transferred to a polyvinylidene fluoride membrane (Millipore). The membrane was incubated in 5% skimmed milk in Tris-buffered saline with 0.2% Tween 20 (TBST) at room temperature for 1 h, and probed with primary antibodies overnight at 4 °C: rabbit anti-occludin (1:1000, Abcam) and monoclonal rabbit anti-β-actin was (1:2000, Abcam). The signals were quantified with an Odyssey Infrared Imaging System (LI-COR Biosciences, Lincoln, NE, USA) and analyzed by ImageJ.

### Statistical analysis

Statistical analysis was performed by a blinded investigator using IBM SPSS Statistics v.19 (SPSS Inc., Chicago, IL, USA). Values are expressed as mean ± s.d., median (quartile) or rate as appropriate. Mean body weight, blood glucose, PT, neurological scores and EB content were analyzed using unpaired *t*-test; infarct volumes, occludin levels and the activity of MMP-9 were compared with one-way ANOVA; HT scores and HT volumes were analyzed with Kruskal-Wallis ANOVA; and mortality rate was evaluated by Chi-square test. Differences with a probability value of *P* < 0.05 were considered statistically significant.

## Results

### Baseline characteristics

**Figure 1:**
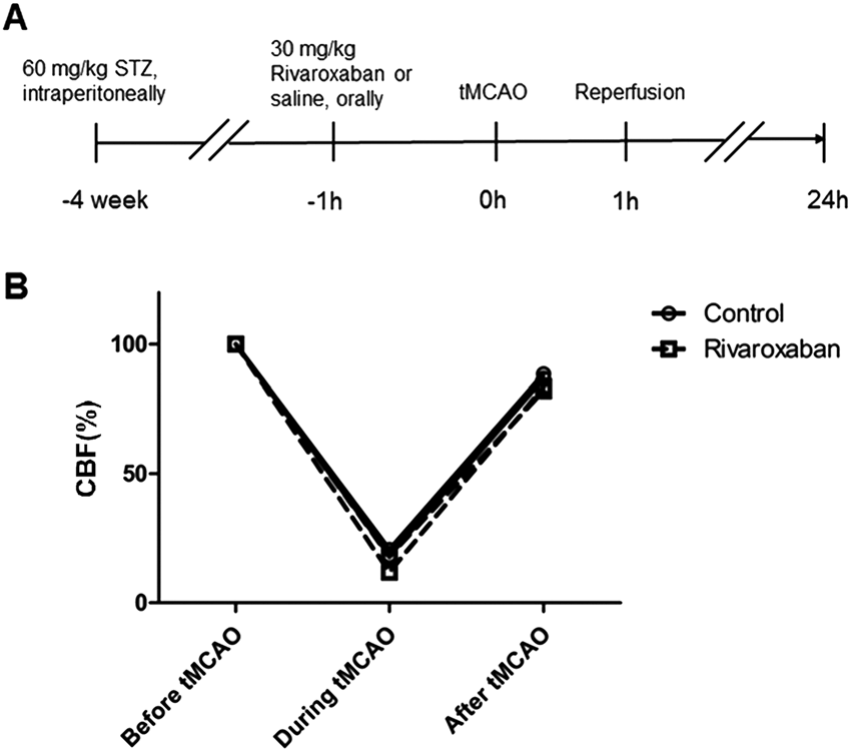
CBF of diabetic rats during ET surgery. (**A**) The diabetes and ET tMCAO model in rats. Rats received 60 mg/kg streptozotocin (STZ) intraperitoneally. Four weeks later, the rats received 30 mg/kg Rivaroxaban or saline orally. (**B**) Laser Doppler measurements during tMCAO surgery in diabetic control rats (n = 33, full line) and Rivaroxaban-treated diabetic rats (n = 33, dash line).

Rats’ body weight was significantly lowered 4 weeks after STZ injection (from 230.45 ± 3.75 g to 199.50 ± 4.23 g of the Rivaroxaban group, *P* < 0.01; and from 229.89 ± 3.52 g to 197.15 ± 3.86 g of the Control group, *P* < 0.01). Mean body weight and blood glucose before ET surgery were not significantly different between the Control and Rivaroxaban group (Table 1). CBF immediately declined to <30% of basal levels during tMCAO, which was recovered to basal levels in both groups. There was no significant difference in CBF before tMCAO, during tMCAO, or at reperfusion between the Control and Rivaroxaban groups (Fig. 1). PT was significantly increased in the Rivaroxaban group (15.68 ± 0.45s before tMCAO and 16.59 ± 0.54s after tMCAO, *P* < 0.01; Table 1).

**Table 1:** Physiological parameters in two experimental groups.

	Rivaroxaban (n = 33)	Control (n = 33)	*P*-value
Body weight (g)	199.50 ± 4.23	197.15 ± 3.86	0.69
Blood glucose before ET surgery (mmol/L)	32.84 ± 0.46	32.27 ± 0.41	0.33
PT before filament insertion (s)	15.68 ± 0.45	10.23 ± 0.14	<0.01
PT after filament extraction (s)	16.59 ± 0.54	10.22 ± 0.12	<0.01
Mortality rate (%)	9.09	9.09	1.00

PT, prothrombin time.

### Neurological score and outcomes

**Figure 2:**
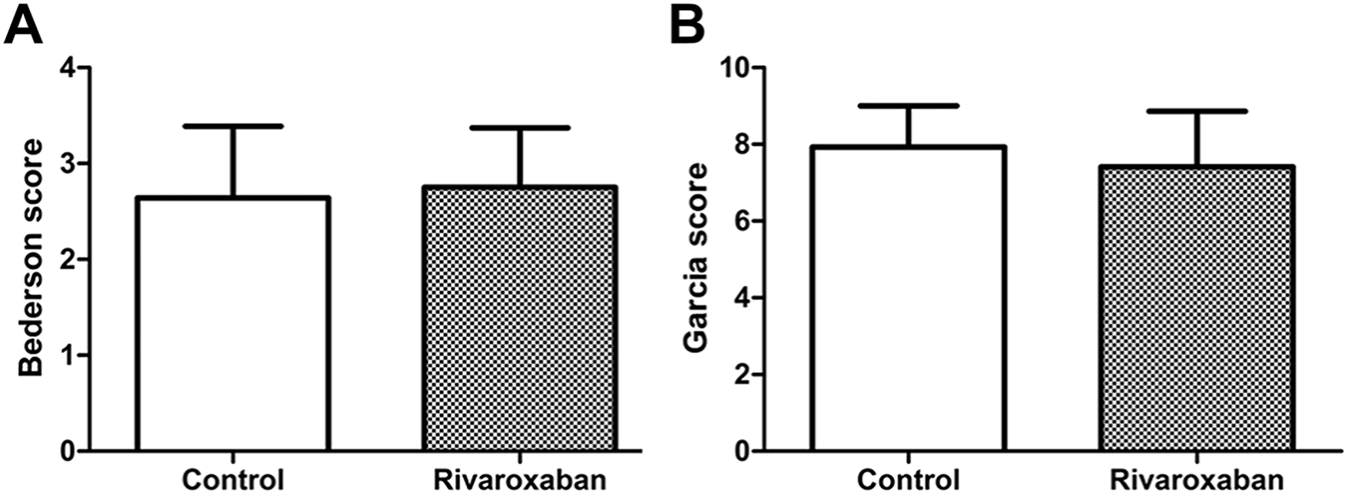
No difference in Neurological outcome under Rivaroxaban treatment post-ET. (**A**) Bederson scale score in 30 mg/kg Rivaroxaban-pretreated rats (n = 12) is 2.75 ± 0.18 and diabetic control rats (n = 12) is 2.64 ± 0.20, without significant difference (unpaired *t*-test, *P* = 0.70). (**B**) Garcia scale score in 30 mg/kg Rivaroxaban-pretreated rats (n = 12) is 7.42 ± 0.42 and diabetic control rats (n = 12) is 7.93 ± 0.29, not significantly different (unpaired *t*-test, *P* = 0.31).

The neurological score did not differ significantly in the Rivaroxaban group than the Control group (2.75 ± 0.18 versus 2.64 ± 0.20 for Bederson scale score, *P* = 0.70; 7.42 ± 0.42 versus 7.93 ± 0.29 for Garcia scale score, *P* = 0.31; Fig. 2A,B).

Two rats died owing to anesthetic accident and 1 due to subarachnoid hemorrhage in both the Control and Rivaroxaban groups, which were excluded from statistical analysis. The mortality rate of both the Rivaroxaban and Control groups was 9.09% (*P* = 1.00; Table 1).

### Infarct volume and HT assessment

**Figure 3:**
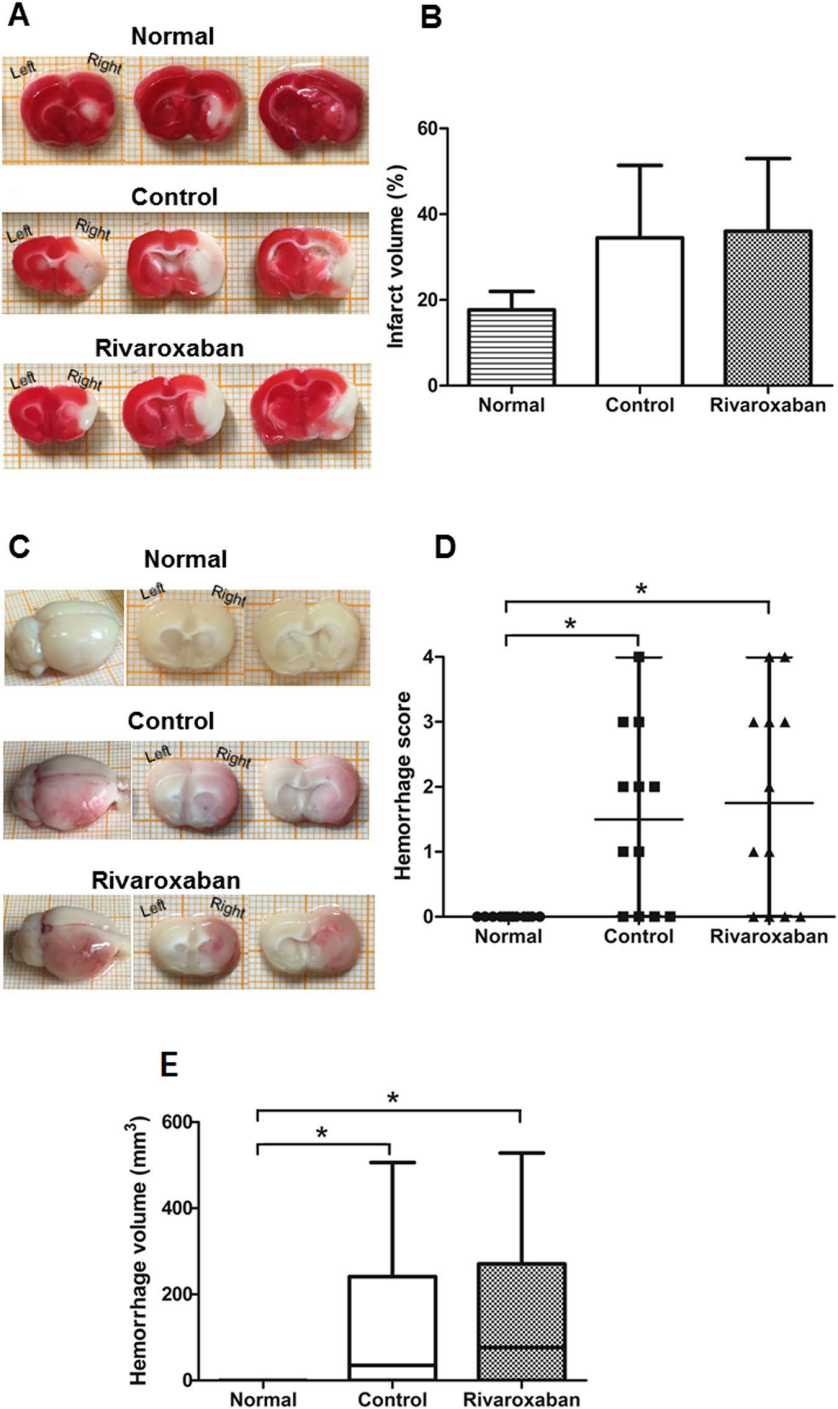
Diabetic rats show increased infarct volume and HT post-ET than normal rats; Rivaroxaban does not increase infarct volumes and HT scores in diabetic rats post-ET. (**A**) Representative images showing 2,3,5-triphenyltetrazoliumchloride (TTC) staining for infarct volume in normal rats (n = 6), diabetic control rats (n = 6) and Rivaroxaban-pretreated rats (n = 6). (**B**) Bar graph depicting infarct volume in all the groups. Ischemic lesion size of the normal group is 17.67 ± 1.76%, the diabetic control group is 34.48 ± 6.90% and the Rivaroxaban group is 36.04 ± 6.92% 24 h after 1 h tMCAO (one-way ANOVA, *P* = 0.07). (**C**) Representative images showing visible hemorrhage in normal (n = 12), diabetic control (n = 12) and Rivaroxaban-pretreated rats (n = 12). (**D**) Macroscopic hemorrhage scored by a masked rater. Individual scores and bars indicate median and range. HT score of normal group, diabetic control group, and Rivaroxaban group is 0 (IQR: 0–0), 1.5 (IQR: 0–2.75), and 1.5 (IQR: 0–3), respectively, at 24 h post-ET surgery. Both the Control and Rivaroxaban groups have higher HT score than the Normal group (*P < 0.05), while the Control and Rivaroxaban group have similar HT score (*P* = 1.00) (Kruskal-Wallis ANOVA and a Dunn-Bonferroni test for post hoc comparisons). (**E**) Box-plot depicting HT volume of rats in all the groups. Median of HT volume of the Normal, Control, and Rivaroxaban group group is 0 mm^3^ (IQR: 0–0 mm^3^, n = 12), 35.1 mm^3^ (IQR: 0–241.20 mm^3^, n = 12) and 76.8 mm^3^ (IQR: 0–270.53 mm^3^, n = 12), respectively, at 24 h after 1 h tMCAO. Both the Control and Rivaroxaban groups have larger HT volume than the Normal group (*P < 0.05) while the Control and Rivaroxaban group have similar HT volume (*P* = 1.00) (Kruskal-Wallis ANOVA and a Dunn-Bonferroni test for post hoc comparisons).

The infarct volumes and HT between the normal, diabetic control and Rivaroxaban-pretreated rats were compared. No statistical significance in infarct volume was found among the three groups (one-way ANOVA, *P* = 0.07). tMCAO for 1 h led to an ischemic lesion size of 36.04 ± 6.92% of the Rivaroxaban group, which was similar to that of the Control group (34.48 ± 6.90%, *P* = 0.85). However, diabetic control rats (*P* = 0.06) and Rivaroxaban-pretreated rats (*P* = 0.04) showed an increased infarct volume 24 h post-reperfusion than normal rats (17.67 ± 1.76%), although a statistical significance was not reached (Fig. 3A,B).

No HT was found in normal rats while 8 of the 12 diabetic control rats and 8 of the 12 Rivaroxaban-pretreated rats displayed HT (*P* < 0.01). Median HT score of the Control group was 1.5 (IQR: 0–2.75) and that of the Rivaroxaban group was 1.5 (IQR: 0–3) 24 h post-ET surgery. Compared to the Control group, pretreatment with Rivaroxaban (30 mg/kg) did not significantly increase the HT score (*P* = 1.00; Fig. 3C,D). Image analysis was used to quantitatively evaluate the HT volume in rats because similar to Tejima’s situation^38^, the type of HT that we observed in the ischemic core was consistently hemorrhagic infarction, not parenchymal hematoma, so we think this method of assessing HT is reasonable. Results of image analysis also showed that HT volume increased significantly in the Control (Median 35.10 mm^3^, IQR: 0–241.20 mm^3^, *P* < 0.05) and Rivaroxaban groups (Median 76.80 mm^3^, IQR: 0–270.53 mm^3^, *P* = 0.03) when compared with the Normal group (Median 0 mm^3^, IQR: 0–0 mm^3^), but did not differ significantly between the Control and the Rivaroxaban group (*P* = 1.00) (Kruskal-Wallis ANOVA followed by Dunn-Bonferroni test; Fig. 3E).

### BBB Permeability, occludin level and MMP-9 activity

**Figure 4:**
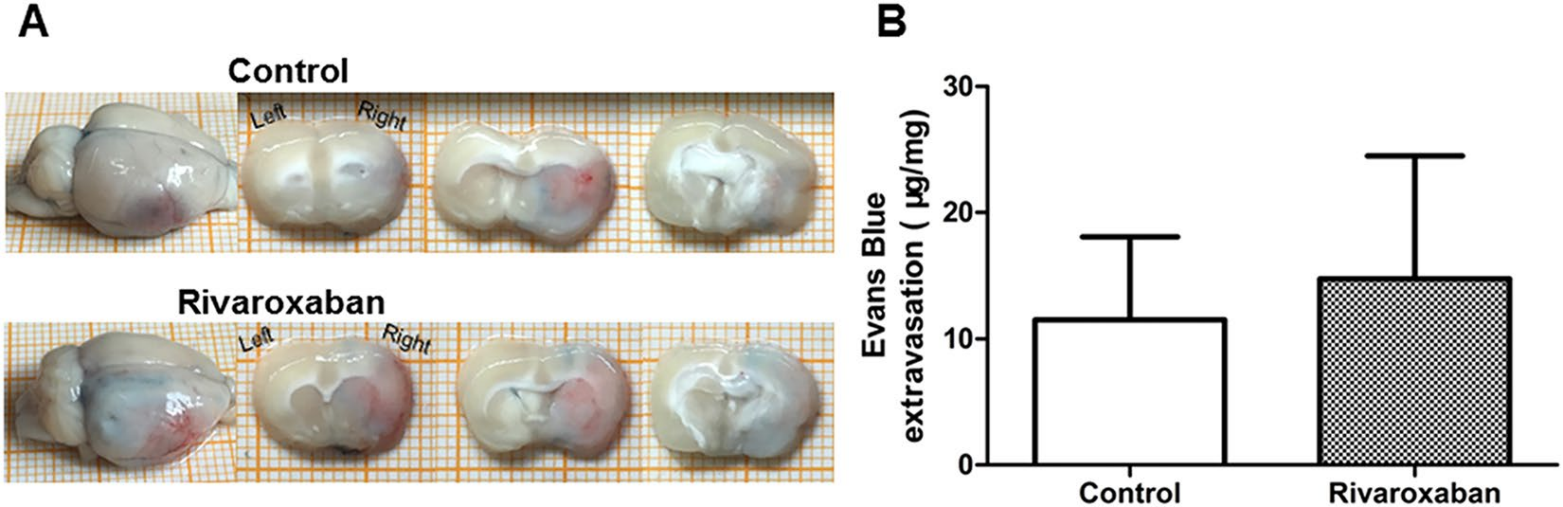
Effects of Rivaroxaban on BBB permeability in rats. (**A**) Topography and extent of EB extravasation in Rivaroxaban-pretreated (n = 6) and diabetic control rats (n = 6). (**B**) Bar graph depicting EB extravasation in all the groups. EB extravasation of the Control group (15.67 ± 3.36 μg/mg) and Rivaroxaban group (16.00 ± 3.18 μg/mg) is similar at 24 h post-ET surgery (unpaired *t*-test, *P* = 0.94).

EB extravasation was similar in the Control (15.67 ± 3.36 μg/mg) and Rivaroxaban groups (16.00 ± 3.18 μg/mg) (*P* = 0.94) (Fig. 4A,B).

**Figure 5:**
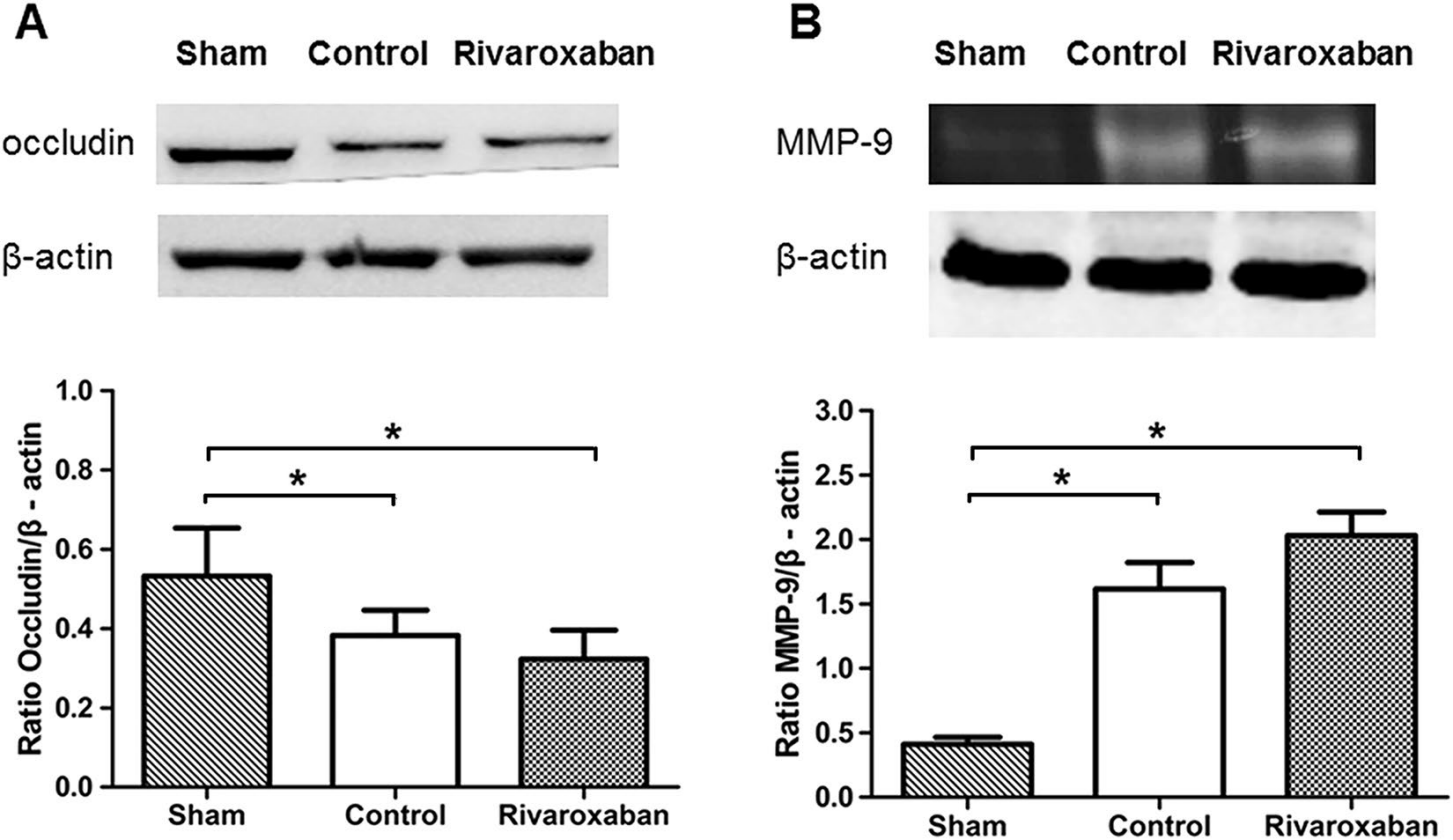
Effects of Rivaroxaban on occludin expression and MMP-9 activity in rats. (**A**) A representative western blot showing changes in occludin expression (n = 6 in each group). Both the Control and Rivaroxaban groups have significantly lower occludin expression level than the Sham group (*P < 0.05), while the Control and Rivaroxaban group have similar occludin expression level (*P* = 0.28) (one-way ANOVA and a LSD test for post hoc comparisons). (**B**) A representative zymogram showing changes in MMP-9 activity (n = 6 in each group). Both the Control and Rivaroxaban groups have significantly higher MMP-9 activity than the Sham group (*P < 0.05), while the Control and Rivaroxaban group have similar MMP-9 activity (*P* = 0.08) (one-way ANOVA and a LSD test for post hoc comparisons).

Occludin expression level was 0.53 ± 0.04, 0.39 ± 0.02, and 0.34 ± 0.03 respectively in the Sham, Control, and Rivaroxaban groups; compared with the Sham group, occludin was significantly lower in the Control (*P* < 0.01) and Rivaroxaban groups (*P* < 0.01); however, the Control and the Rivaroxaban group had similar occludin expression level (*P* = 0.28) (one-way ANOVA followed by LSD test; Fig. 5A) (Also see Supplementary File).

MMP-9 activity was 0.41 ± 0.03, 1.61 ± 0.04, and 1.77 ± 0.09 respectively in the Sham, Control, and Rivaroxaban groups. Both the Control (*P* = 0.01) and Rivaroxaban groups (*P* < 0.01) showed significantly higher MMP-9 activity than the Sham group while the Control group and the Rivaroxaban group had similar MMP-9 activity (*P* = 0.08) (one-way ANOVA followed by LSD test) (Fig. 5B) (Also see Supplementary File).

## Discussion

Our study proposes 2 new findings: (1) Rivaroxaban does not increase the risk of HT in experimental cerebral ischemia and ET in diabetic rats. (2) Rivaroxaban does not increase BBB permeability post-ET for proximal MCA occlusion in diabetic rats.

Previous studies used tMCAO rodent model to simulate acute ischemic stroke (AIS) patients with rt-PA recanalization. However, the clinical relevance was questioned due to distinct CBF profiles upon reperfusion between tMCAO (abrupt reperfusion) and alteplase treatment (gradual reperfusion), resulting in differing pathophysiologies. Thus, the tMCAO model is an ET model^31^.

This and previous studies have consistently demonstrated that anticoagulation with Rivaroxaban will not increase HT with or without thrombolysis in various models of focal cerebral ischemia and recanalization in mice and rats^11,12^, similar to the human situation^41^. AHA/ASA AIS guidelines (2015) pointed out that the benefit of ET in stroke patients under anticoagulation is unclear^42^ and hitherto, only one case of 83-year-old woman treated by both rt-PA and ET under Rivaroxaban treatment was reported^43^. The author concluded that thrombolysis and/or ET might be safe for patients treated with the new anticoagulant Rivaroxaban. Meanwhile, the safety of Rivaroxaban in diabetic patients is unclear. We, firstly, established a model of diabetes and ET during oral anticoagulation with Rivaroxaban. Herein, we concluded that anticoagulation with Rivaroxaban does not cause an excess risk of HT in diabetes post-ET.

To induce Rivaroxaban-associated ICH, three doses (3, 10, and 30 mg/kg) were used in the preliminary experiment. The results showed that 30 mg/kg could increase the PT by 2–3-fold, and the drug’s action lasted for >4 h. Zhou *et al*.^9^ demonstrated a similar phenomenon. Since the dose is >30-fold higher than that in clinical practice^44^, these findings suggest that even very high doses of Rivaroxaban cannot increase the risk of HT in diabetic rats post-cerebral infarction and ET, and confirmed the safety profiles of this drug.

The emerging neurovascular unit concept underlies the important contribution of cerebral blood vessels to the pathophysiology of stroke^45^. Cerebrovasculature is susceptible to damage by hyperglycemia^19^. Vascular injury and potentially subsequent vascular dysfunction in diabetes may be as important as the neuronal injury in determining neurological deficit. Diabetes cerebrovascular remodeling is characterized by increased vessel tortuosity, vascular endothelial growth factor (VEGF) expression, and MMP-2, and -9 activity^46^. Newly formed or remodeled cerebrovasculature vessels cannot resist the impact of ischemia/reperfusion injury and increased bleeding occurs. In other words, VEGF secretion and angiogenesis led to HT in diabetes. Hollborn *et al*.^47^ found that the activated blood coagulation factor Xa induced chemotaxis of retinal pigment epithelial cells and stimulated the release of angiogenic growth factors such as VEGF. So, inhibition of factor Xa may have a direct effect on VEGF secretion and suppress coagulation-induced angiogenesis. It can partially explain our result of no extravagant HT in Rivaroxaban-treated diabetes experimental cerebral ischemia and ET.

Upregulation of MMPs may be a common link between diabetes and IS pathologies. In IS, increased MMP-9 disrupts basal lamina and neurovascular unit structure promoting edema and HT^48–50^. MMP-9 knockout mice are protected against IS^19,51^. In diabetes, the MMP system is dysregulated and vascular wall integrity may be weakened, setting the stage for an aggravated damage during stroke. Cerebrovascular permeability increases in diabetes because of diabetes-induced damage to the BBB function and/or because of the immature nature of the newly formed vessels^52^. Tight junction proteins such as occludin and claudin-5 have essential roles in regulating BBB stability^53^, and endogenous overexpression of MMP-9 in brain endothelial cells results in significant degradation of tight junction proteins occludin and claudin-5^54^. Our result accords with the opinion that MMP-9 expression increases significantly in diabetes post-IS. Furthermore, Rivaroxaban may have little effect on MMP-9 expression. This study also reveals that Rivaroxaban does not increase occludin degradation and has no effect on BBB stability in diabetes post-experimental ET, which is similar to normal experimental stroke models^55^.

Limitations: In this study, HT was designed to be assessed by a scoring method and spectrophotometric measurement of hemoglobin concentration. However, as the HT in both the experimental and Control groups was hemorrhagic infarction, the optical density could not be detected at such a low level; therefore, the results were not reported here. Ploen *et al*.^12^ reported that even treated with rt-PA, the hemoglobin concentration of HT in 30 mg/kg Rivaroxaban-pretreated mice was still very low (0.68 μL). In this situation, we used image analysis as an alternative to evaluate the HT volume quantitatively and also found that HT volume did not differ significantly between the Control and Rivaroxaban group.

Our results provide evidence that Rivaroxaban does not increase HT post-ET in diabetes rats. Fewer effects of Rivaroxaban on post-ischemic BBB permeability may be partially explanatory. The clinical application of NOACs (Rivaroxaban) not only can reduce the incidence of AF-related IS but also may not affect ET treatment once AIS happens.

## Electronic supplementary material

Supplementary images
