# Supplementary material for: Rivaroxaban does not influence hemorrhagic transformation in a diabetes ischemic stroke and endovascular thrombectomy model
Source: Sci Rep. 2018 May 9;8:7408. doi: 10.1038/s41598-018-25820-y (PMC5943582; doi:10.1038/s41598-018-25820-y)

Supplementary Information (full-length images)

Rivaroxaban does not influence hemorrhagic transformation in a diabetes ischemic stroke and endovascular thrombectomy model

Feng-Di Liu[a,†], Rong Zhao[a,†], Xiao-Yan Feng[b,†], Yan-Hui Shi[a,†], Yi-Lan Wu[a], Xiao-Lei Shen[a], Ge-Fei Li[a], Yi-Sheng Liu[a], Ying Zhao[a], Xin-Wei He[a], Jia-Wen Yin[a], Mei-Ting Zhuang[a], Bing-Qiao Zhao[c,*], Jian-Ren Liu[a,*]

β-actin (2016-4-1)

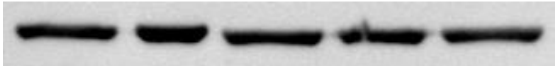

occludin (2016-4-8)

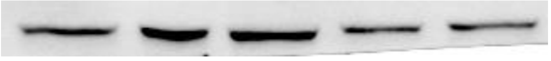

Full-length blots (2016-4-8): HIF & occludin

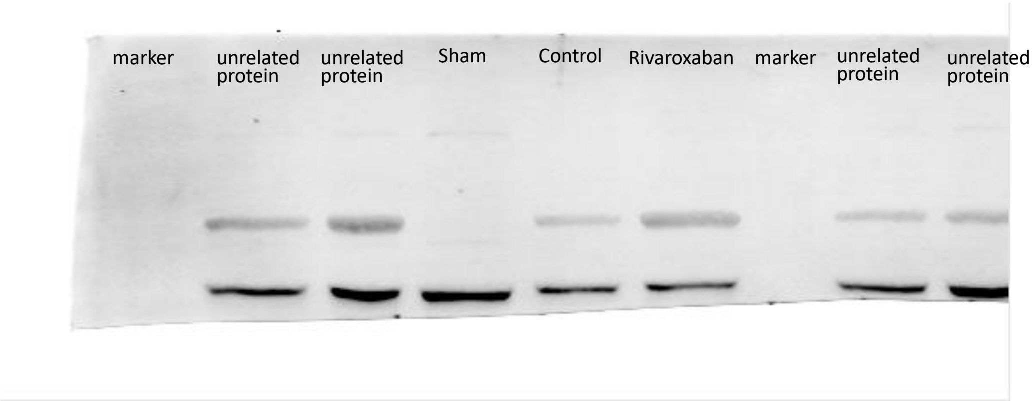

β-actin (2016-1-21)

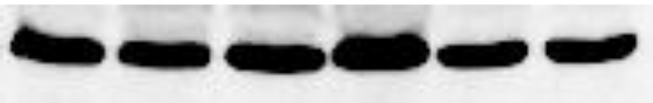

Full-length gel (2016-1-27): MMPs

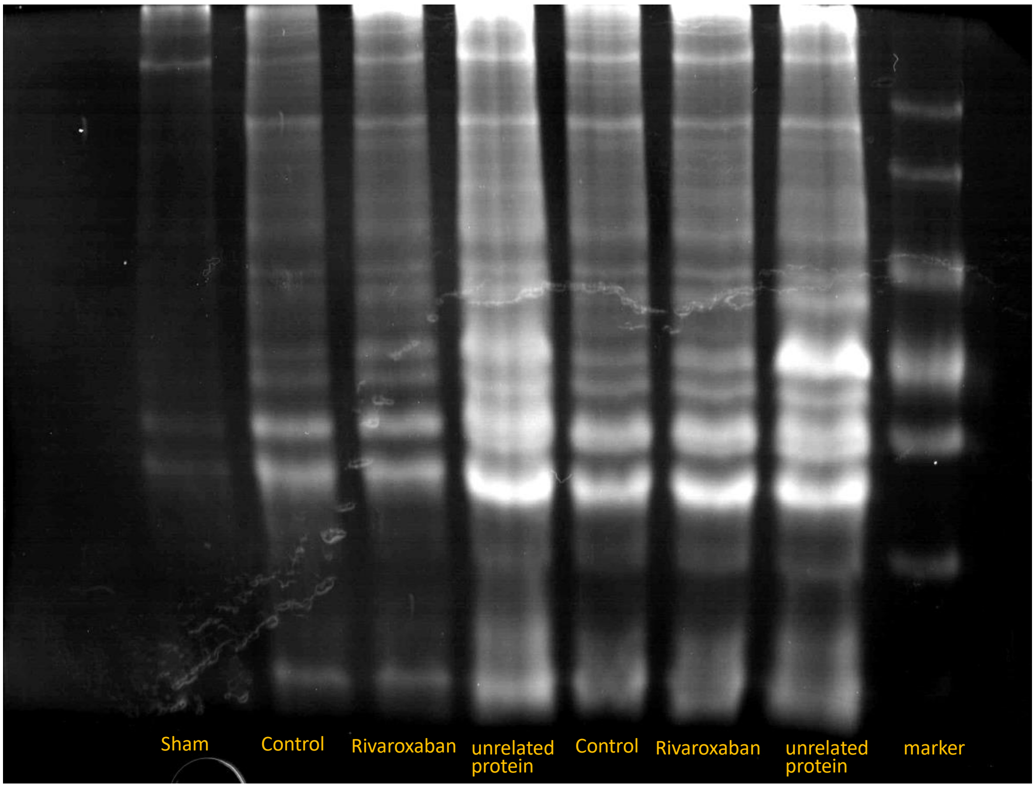

Supplement: Supplementary file 1 — Supplementary images [file 41598_2018_25820_MOESM1_ESM.pdf]
